# The Patient with Difficult Cancer Pain

**DOI:** 10.3390/cancers11040565

**Published:** 2019-04-19

**Authors:** Sebastiano Mercadante

**Affiliations:** Main regional center for pain relief and supportive/palliative care, La Maddalena Cancer center, via San Lorenzo 319, 290146 Palermo, Italy; terapiadeldolore@lamaddalenanet.it or 03sebelle@gmail.com

**Keywords:** cancer pain, opioids, neuropathic pain, incident pain, cognitive disturbance, addiction, psychological factors

## Abstract

Most patients with cancer pain can be managed with relatively simple methods using oral analgesics at relatively low doses, even for prolonged periods of time. However, in some clinical conditions pain may be more difficult to manage. Various factors can interfere with a desirable and favorable analgesic response. Data from several studies assessing factors of negative pain prognosis have indicated that neuropathic pain, incident pain, psychological distress, opioid addiction, and baseline pain intensity were associated with more difficult pain control. In this narrative review, the main factors that make the therapeutic response to opioids difficult are examined.

## 1. Introduction

Pain is a highly prevalent and distressing symptom that represents a major health problem in cancer patients. The incidence of pain ranges from 24% to 60% and approaches 58% to 69% in those receiving active treatment and who are in the advanced stage of disease, respectively. Moreover, pain is still present in more than 30% of patients after curative therapy [1]. Opioids represent the mainstay of the pharmacological treatment of cancer pain. Cancer pain can be controlled in most patients by appropriate use of opioids that maintains low doses of opioids for prolonged periods of time. However, there is a huge variability in opioid responsiveness across patients. A percentage of patients, quantified as 10–20%, will experience pain which requires more complex approaches [2]. Analgesics treatments may lose efficacy because dose escalation is not followed by a favorable analgesic response and the therapeutic window is restricted by the development of side effects, despite using symptomatic drugs. A gradual increase in dosage that is not followed by a change in analgesic improvement should suggest that treatment is inefficient, and possibly is creating the conditions for increased neuronal excitability, a phenomenon known as opioid-induced hyperalgesia [3].

The need for rapid increases of opioid dosage may be due to the progression of disease or the presence of a masked problem affecting the dose/response relationship. In the last decade, research has focused on opioid responsiveness to identify the possible factors capable of influencing an unfavorable response to opioids. The opioid response is variable and extends from conditions where patients achieve a good analgesia that is maintained even for prolonged periods of time with minimal changes in the opioid dosage, to conditions where patients are completely unresponsive and cannot achieve an adequate analgesia unless the dosage is at a level that it is producing intractable adverse effects that are not responsive to symptomatic therapy [4]. Therefore, the ceiling effect of opioids is determined by the dosage associated with the occurrence of adverse effects which limits further dose increases. Both analgesia and adverse effects are observed with different opioid dosages among patients, depending on several factors that make each patient unique.

Generally, opioid responsiveness has been defined as the degree of analgesia that can be obtained after an optimization of the increase in the dosage of an opioid until adequate analgesia is achieved or, alternatively, side effects occur. Various factors can interfere with the desirable favorable analgesic response, and are represented by disease progression, development of tolerance, the tendency to develop side effects, pain mechanisms, the presence of some active or toxic metabolites, pharmacokinetics, and pharmacodynamics, as well as genetic factors [5].

Of interest, the general response to opioids cannot be considered definitive after a treatment with a single opioid. The substitution of the opioid and/or route of administration has become a very effective approach for improving the performance of opioid treatment, overcoming the traditional concept of general responsivity to opioids. It is based mainly on a different characterization of opioids, which are apparently similar, and able to determine a different clinical response in the same patient [6].

Different factors play a role in opioid response. Any process capable of reducing the efficacy of the current analgesic regimen may influence the opioid responses: the development of tolerance, the individual propensity to develop adverse effects that are poorly responsive to symptomatic treatment, the pain mechanism and temporal pattern of pain, the metabolic variabilities influencing pharmacokinetics, the genetic predetermination, and the psychological distress. Moreover, the advanced stage of disease and associated multiple organ failure, may render the clinical presentation more complex.

Data from several studies assessing factors of negative pain prognosis have indicated that younger age, neuropathic pain mechanism, a subtype of breakthrough pain associated with movement due to bone metastases, psychological distress, and high levels of baseline pain intensity may be associated with greater difficulties in controlling pain. Other possible variables involved include sleep, opioid dose, and addiction [7,8,9]. This narrative review will assess the problems that challenge the analgesic treatment.

## 2. Incident Pain

Incident pain is a subtype of breakthrough cancer pain (BTcP). BTcP has been defined as a transient peak of pain that occurs either spontaneously or is triggered by a recognizable cause, despite a stable and well controlled basal pain [3]. In a recent study about 40% of patients with BTcP presented predictable incident pain, prevalently triggered by movement, due to bone metastases [10]. This predictable condition is observable in patients with bone metastases in which movement produces a transient peak of pain, to which they frequently react, limiting their activities to avoid this peak of pain intensity, that is otherwise of mild intensity or absent at rest. Consequently, this subtype of BTcP strongly interferes with quality of life preventing the physical activity. This condition of a transitory exacerbation of pain induced by even minimal physical activity, prevalently in subjects with metastatic bone diseases, is very challenging because it is difficult to optimize the opioid dosage to control both background pain and incident pain. Any attempt to increase the dosage to prevent the occurrence of incident pain is often associated with adverse effects when the patient is at rest. This condition is challenging for the pain physicians.

## 3. Neuropathic Pain

Neuropathic pain is a major issue in cancer pain management. Difficulties in understanding the underlying mechanisms of neuropathic pain have been invariably reported. In fact, there is no validated and universally accepted clinical criteria diagnosing neuropathic pain in cancer patients. Neuropathic pain includes so many heterogeneous conditions, which present different etiologies, that it makes a classification difficult and limited for a possible underlying cause or the anatomical site [11].

Neuropathic pain has been considered a negative pain predictor factor. From a clinical point of view, neuropathic pain determines cellular biochemical modifications which resemble those described for tolerance. In these situations, there is a common cellular cascade involving the activation of *N*-methyl-d-aspartate receptors, which results in a change of the dose-response curve to morphine with a tendency to the development of a phenomenon named hyperalgesia. This is characterized by a series of biomolecular events for which, theoretically, an opposing force stimulates an increase in the dosage to achieve an equal analgesia. In other words, it is as if opioid tolerance would be present in the absence of a previous opioid exposure [5]. It has been observed that neuropathic pain is more frequently associated with the use of strong opioids, adjuvants, and alterations in the cognitive, functional, and social level [12]. The clinical response to opioids in patients with neuropathic pain has been considered a controversial topic. Neuropathic pain in cancer patients has been consistently described as a negative prognostic factor associated with a decrease in opioid response. However, neuropathic pain does not result in an inherent resistance to opioids, but it may be considered as a prognostic factor limiting a favorable outcome [3]. Other studies assessing the pain prognostic factors have shown that neuropathic pain may require more aggressive pain treatments, which are associated with a larger use of adjuvants and high opioid dosages [3,7,8]. From a clinical perspective, a change in opioid response depends on the actual context and a specific kind of nerve damage. In a recent study, patients achieved analgesia in conditions of “definite neuropathic pain”, although they required a more aggressive and complex pain management with careful utilization of opioids, including the opioid switching, and the use of adjuvant drugs with a specific effect on neuropathic pain [13].

Although patients with neuropathic pain are likely to require higher doses of opioids to obtain an acceptable analgesia, which may be associated with more adverse effects, neuropathic pain should be considered inevitably resistant to opioids [8,13].

On the other hand, the response obtained with one opioid cannot be generalized to other opioids. In fact, the use of an alternative opioid in cancer patients who are refractory to previous opioids, is often successful. Features from the Edmonton Classification System for Cancer Pain were found to be not absolutely predictive of pain management complexity, at the follow-up visit and after a palliative care consultation. However, the presence of neuropathic pain was associated with less chance of achieving a personalized pain goal [14].

## 4. Psychological Distress

Psychological factors have been reported to be associated with a poorer response to opioid treatment. Patients with a profile of depressive mood and anxiety reported more symptoms of varied intensity [15]. In a large sample of patients with advanced cancer, depression was found to be independently associated with many somatic symptoms [16]. In another study performed in patients admitted to hospice care, depression and dyspnea were independently associated with anxiety [17]. In a secondary analysis, anxiety and depression were strongly and independently associated with mental health domains and a higher burden of somatic symptoms. Depression was more intensively associated with multiple life domains [18]. Similar findings were reported in another secondary analysis in which the level of depression severity significantly correlated with many physical symptoms and their severity, regardless of cancer diagnosis, functional status, anticancer therapies, and survival [19]. Finally, these psychological symptoms have been found to be associated with prolonged hospitalization [20]. Thus, the presence of anxiety and depression in advanced cancer patients may concur to symptom hyper-expression and possibly could make their treatment challenging. In particular, it has been reported that pain may be overexpressed in patients with high levels of anxiety. Indeed, anxiety and depression have been frequently found to coexist and influence each other [21].

## 5. Cognitive Status

Delirium is a neuropsychiatric condition which has frequently been reported in cancer patients in advanced stages of disease. The causes of this condition often are multifactorial. It may result in significant distress for the patient, their family, as well as health caregivers. Delirium promotes the functional decline, increases the length of hospital stay and medical cost, and is associated with short survival [22,23,24]. Indeed, delirium is one of the most frequent indications for palliative sedation in the last days of life, either at home or in patient units [25,26,27,28]. This disorder has been variably reported in the literature, ranging between 13% and 88% in the palliative care population, particularly at the end of life. In a study conducted in many patients admitted to different palliative care settings, about 42% of patients presented a status of delirium [29]. One week after palliative care this percentage increased (about 67%). However, in patients admitted to an acute palliative care unit there was a significant decrease in the number of patients with delirium after one week of palliative care [30]. These differences are possibly due to a more specialized intervention or simply because this group of patients were seen in an earlier stage of disease.

This altered cognitive function may lead to a misinterpretation of symptom assessment and may lead to inappropriate interventions. Regrettably, this neuropsychiatric disorder is often unrecognized or insufficiently diagnosed, and poorly managed by clinicians [24,31,32]. Delirium impairs recognition of physical symptoms and complicates optimal symptom management [33,34]. A retrospective study showed that patients who developed delirium during admission reported a higher level of symptoms, particularly pain [34].

An inappropriate assessment of the cognitive status in cancer patients may cause severe iatrogenic problems with undue opioid dose escalation [34]. Changes in the cognitive function are associated with rapid escalation of the opioid dosages [23]. The presence of cognitive alterations has already been described as a possible factor leading to hyperexpression of pain intensity, and therefore resulting in a more delicate condition, requiring expertise in concomitantly treating delirium and pain [35,36].

## 6. Pain Intensity

Patients who experience moderate to severe pain have been reported to require a longer time to achieve an acceptable level of pain. Patients who had higher levels of pain at an imprecise time of assessment have been reported to require higher opioid doses [37]. However, the reason of this finding probably relies on a non-homogeneous analgesic approach that biased the outcome. For example, opioid dose escalation was performed over a prolonged period, which renders the data unreliable. It is quite evident that in a temporal context, pain intensity may change during the different phases of treatment, also considering the timing of interception of the patient along the disease trajectory and a correct concomitant pain management. It is more likely that a prolonged period of undertreatment may result in more distress and more difficulties in achieving pain control [38,39]. In an acute palliative care unit, where more aggressive approaches of opioid dose titration were available, there was no correlation between the level of baseline pain intensity recorded at admission and the analgesic response [13]. In some studies, the baseline pain intensity, that is the level of pain intensity at time of the visit, has been found to worsen the outcome, resulting in a negative factor for pain prognosis [9,37,40,41]. In all these studies, however, it was evident that what resulted was a clear clinical undertreatment, which strongly biased the outcome. Indeed, the very long time to stabilize patients’ conditions, ranging from eight to 22 days, suggested non-standardized and non-optimized methods for opioid dose titration. In other cases, the retrospective long follow up of outpatients (three weeks), with only one therapeutic intervention, would have biased the outcome. On the other hand, the concept of pain intensity is a dynamic issue, as it depends on the moment the patient is seen and if the patient was properly treated. Of interest, recent studies are consistent with the daily practice in experienced palliative care centers where an acceptable level of pain intensity is achieved in a few days in most patients using an adequate opioid regimen individually planned [13,38]. Recently, it was reported that the higher the initial pain intensity, the better a patient’s impression of overall improvement or achievement of the personalized goal [42]. This could be explained by the feeling of improvement perceived by patients when they perceive a rapid and evident decrease in pain intensity after expert palliative care management, and also because their level of expectation is more easily reached with a small decrease in pain intensity. In other words, patients who start with a high level of pain intensity have lower expectations and are more likely to achieve an adequate level of analgesia, as well as experience better satisfaction [42,43]. Data from this study confirms that the higher the initial pain intensity, the better the patient satisfaction after an appropriate pain management. 

## 7. Aberrant Behaviors

Somatization has been defined as the process by which a psychological distress results in a hyperexpression of physical symptoms. It seems that patients reporting addictive behaviors may have a greater risk for chemical coping and somatization. Opioids may produce a double effect precipitating drug-seeking behavior. Chemical coping develops when patients use medications like opioids that are not properly prescribed, or they misuse them to cope with stressful life events. The use of not-prescribed opioids can lead to their misuse and subsequently to serious complications including neurotoxicity, respiratory depression, or even death. An association has been found between chemical coping and opioid dose escalation and pain expression. Moreover, patients with aberrant behavior for alcohol or drugs can suffer because of a stigmatization on behalf of health care professionals. This attitude may result in a further undertreatment of cancer pain [44]. Patients screened for alcohol have been found to be referred earlier to palliative care, exhibited higher symptom expression, and have more frequently received opioids. 

About 18% of patients with advanced cancer who were visited by a palliative care team were diagnosed as having chemical coping. Of interest, only 4% of these patients had this aspect documented in their medical records. Alcoholism, younger age, better performance status pain, as well poor well-being were associated with chemical coping [45].

Smoking status and alcohol or illicit drug use may play a role in the symptom expression of advanced cancer patients. A smoking history may be a risk for opioid misuse. Former or current smokers were likely to have an alcohol history and illicit drug use. Indeed, current smokers showed a higher level of pain, as they would hyper-express this symptom [46,47]. Alcoholism has been found to be associated with a history of smoking and illegal drug use, placing these patients at risk for abuse and inappropriate opioid escalation [48]. 

Patients who were current and former smokers had higher levels of pain intensity, required higher opioid doses, and were at more risk for the use of non-prescribed opioids [49]. This data suggests a need for closer monitoring and proper psychosocial support of advanced cancer patients who are smokers. 

Despite these observations, data suggesting that smoking, alcoholism or aberrant drug behavior may influence symptom expression and management in advanced cancer patients, remain controversial, and therefore require further studies to be performed in different countries [50].

## 8. Conclusions

There are many problems presented when treating patients with cancer pain having an unfavorable clinical response to opioids. An appropriate assessment of some clinical factors inherent to pain mechanism and disease, as well as other clinical individual factors that may amplify pain expression, could enhance the management of individuals with a tailored treatment. Some patients with specific conditions, once diagnosed, should be referred to a specialist for advice.
